# Pediatricians’ knowledge, attitude and practice on treating children with red eye disease

**DOI:** 10.1186/s12886-022-02755-7

**Published:** 2023-02-13

**Authors:** Dina Mostovoy, Anna Bunin, Yotam Eyni, Merav Ben Natan

**Affiliations:** 1 Department of Ophthalmology, Barzilai Medical Center, Ashkelon, Israel; 2 Ben Gurion University, Beer Sheva, Israel; 3 Pat Matthews Academic School of Nursing, Hillel Yaffe Medical Center, P.O.B. 169, 38100 Hadera, Israel

**Keywords:** Red eye disease, Knowledge, Attitudes, Pediatricians

## Abstract

**Background:**

Pediatricians play an important role in the early detection and prompt treatment of ocular disorders in children, including red eye disease. Our aim was to examine the knowledge level of pediatricians regarding treating children with red eye disease, as well as the factors that affect the knowledge level, and the potential implications of a low level of knowledge.

**Methods:**

In this correlational quantitative study, 152 expert pediatricians completed a questionnaire that included questions on knowledge, attitudes, and experience in treating red eye disease.

**Results:**

Respondents’ mean level of knowledge was moderate. Most of the respondents (89.5%) knew that the most likely diagnosis for a child with red eyes and a discharge is viral conjunctivitis and that pain, blurred vision, and a clouded cornea, are symptoms indicative of a more complex systemic problem. In contrast, 78.3% of the pediatricians claimed that the treatment of choice for viral conjunctivitis is a conservative treatment that includes eye flushing and strict hygiene. However, 14.5% (*n* = 22) of the pediatricians were found to prescribe antibiotics. A negative association was found between the pediatrician’s age and years of experience, and level of knowledge concerning treatment of children with red eye. A strong positive association was found between pediatricians’ level of knowledge and their attitudes to performing eye tests. Moreover, a negative association was found between the level of knowledge and the number of cases in which pediatricians prescribed antibiotics for children with red eye.

**Conclusions:**

The research findings indicate that lack of knowledge was more conspicuous among pediatricians with more experience. Knowledge appears to be critical both for readiness to treat red eye and for proper treatment. It is necessary to provide pediatricians with tools for treating eye disorders in children and to refresh their knowledge on red eye, particularly among pediatricians with more years of experience.

## Background

Red eye is defined as sudden redness that appears on the conjunctiva, eyelids, or adjacent structures. It is one of the most common complaints among children. This symptom is usually perceived by pediatricians as resulting from conjunctivitis, but it is not always associated only with eye disorders [1]. Red eye might be a clinical indication of several systemic diseases that might be evidence of a grave condition. Pediatricians are often the first specialists that meet the children suffering from “red eye” [2, 3].

The pediatrician has a crucial role in reaching decisions that involve examining the severity of eye conditions [1]. A single episode of acute conjunctivitis can cause corneal scars that might affect vision or lead to changes in the conjunctiva that result in a chronic degenerative problem or even blindness [4, 5]. Hence, taking an accurate and extensive anamnesis that includes ruling out trauma, a medical history of previous eye surgery, proper pupil testing, and the child’s alertness, helps diagnose the cause that requires treatment [4].

In such cases involving children, the directives of the American Association of Pediatrics recommend beginning with conservative treatment [3]. Sheikh et al., however, proved that use of topical antibiotics versus placebo hastened microbiological and clinical recovery in the first days of the illness, but noted that in most cases the illness disappeared on its own in any case [5]. Furthermore, use of topical antibiotics was found related to a rise in resistance to antibiotics, discomfort to the patient, higher costs for the parents, and an increase in unnecessary use of medications. Moreover, use of topical antibiotics might result in side effects such as allergic reactions and even anaphylactic reactions, aplastic anemia, urticaria, and many others.

The current recommendations of pediatric ophthalmologists for the treatment of conjunctivitis begin with eyelid hygiene and cleaning the discharge, use of artificial tears, and monitoring. However, according to the literature [6, 7], pediatricians do not often follow this course and tend to prescribe antibiotics.

According to the accepted instructions in the pediatric literature (Nelson textbook of pediatrics [8], UpToDate [9]) there is a difference between viral conjunctivitis and bacterial inflammation, with different treatment recommendations that include conservative treatment for the former and a recommendation of topical antibiotic treatment for the latter, but in practice in most cases they are indiscernible. According to the American Academy of Pediatrics, pediatricians must promptly treat eye disorders such as red eye in children, to prevent lifelong vision disorders. Accordingly, pediatricians must be capable of identifying causes of red eye.

A study conducted in Jordan [6] indicated that pediatricians thought that conjunctivitis is a result of infection, while in Kenya they thought that red eye is a result of trauma. In both studies, patients were mostly referred to an ophthalmologist for further treatment [7]. A study conducted in Ethiopia among physicians also found no unequivocal knowledge and essential understanding of the treatment and causes of red eye [10]. A study conducted in Germany discussed general physicians’ lack of knowledge on the subject.

Since common eye disorders among children are diagnosed and managed by pediatricians, it is important to examine this issue, particularly since certain eye diseases might endanger children’s vision or even be potentially fatal. Hence, the purpose of the current study is to examine the level of knowledge among pediatricians regarding treating children with red eye, as well as factors affecting their level of knowledge and the potential implications of a low level of knowledge.

## Methods

### Research design

A correlational quantitative questionnaire study.

### Respondents

The sample size was calculated based on the results of Wanyama [7], according to which approximately 70% of the pediatricians are expected to have level of knowledge classifiable as poor. It was determined that a sample size of about 200 participants will provide a sufficient sample size to draw assumptions at the 95% confidence interval with a 5% margin of error and 80% power. Only 152 questionnaires were returned fuly completed, for a response rate of 76%.

### Research tool

The research tool was a validated questionnaire built by Wanyama [7], translated into Hebrew and adapted to Israeli reality by the researchers. The tool was reviewed and validated by three ophthalmologists and pediatricians. Its level of reliability is Cronbach’s alpha of 0.80. The questionnaire consists of four parts: The first part relates to sociodemographic and medical experience (4 questions). The second part discusses experience with treating red eye (2 questions). The third part explores level of knowledge (4 knowledge questions). The fourth part explores attitudes to treating children who present with a complaint of redness (3 questions).The questions were multiple choice type.

### Research procedure

After receiving the approval of the Helsinki Committee at the Barzilai Medical Center in Ashkelon, e-mail addresses of pediatricians were obtained from the Israeli Medical Association. All pediatricians in Israel are registered in this association. All methods were carried out in accordance with relevant guidelines and regulations. A request to participate in the study was sent them by e-mail and those who agreed were sent a link to an online questionnaire. Completion of the questionnaire took about 7 min and anonymity was assured. The respondents’ information was collected with no identifying details.

### Statistical analysis

The data were analyzed with the SPSS program, version 26. For categorical variables, frequencies and percentages were examined, for the normally distributed continuous variables, means and standard deviations were calculated. The distribution of the variables was portrayed according to the specific aims. A Pearson test was conducted to examine the associations between the quantitative variables. A t-test for independent samples was conducted to examine the associations between the quantitative and dichotomous variables. The differences were calculated as statistically significant, where *p* < 0.05.

## Results

The participants in this study were *N* = 152 pediatricians with a mean age of M = 45.8 (SD = 11.88) and a range of 27–82. Most were women (*n* = 100, 65.8%), where the mean number of years they had been working as pediatricians was M = 14.77 (SD = 13.42), and most were living in central Israel, 58% (see Table 1).

**Table 1:** Sociodemographic information of the pediatricians (*N* = 152)

	M	SD	n	%
Age	45.8	11.88	152	
Sex			152	
Men			52	34.2
Women			100	65.8
Years in the profession	14.77	13.42		

To examine the knowledge level of the participants, 4 knowledge questions on treating red eye in children were explored. The research findings showed that the respondents’ mean level of knowledge was moderate, M = 3.28 (SD = 0.77, range 1–4).

Most of the respondents (89.5%) knew that the most reasonable diagnosis for a child with red eyes and discharge is viral conjunctivitis, 7.9% thought that it was bacterial conjunctivitis, 2% a systemic disease, and 0.6% an allergy. Most of the respondents (89.5%) knew that pain, blurred vision, and a clouded cornea, are symptoms indicative of a more complex systemic problem.

Of all respondents, 78.3% claimed that the treatment of choice for viral conjunctivitis is a conservative treatment that includes eye flushing and strict hygiene. However, 14.5% (*n* = 22) of the pediatricians were found to prescribe antibiotics. In addition, 71.7% said that they would refer a child with red eye to an ophthalmologist in any case of disrupted or blurred vision.

A negative association was found between the pediatrician’s age and years of experience, and level of knowledge concerning treatment of children with red eye. Namely, the older the pediatrician and the more years of experience, the lower the level of knowledge regarding treating red eye in children. In contrast, no association was found between the number of red eye cases treated by pediatricians and their knowledge (*p* > 0.05) (see Table 2).

**Table 2:** Pearson correlations between level of knowledge, number of years as physician, and number of red eye cases treated

	**Age**	**Number of years as physician**	**Number of red eye cases treated**	**Number of cases for which antibiotics were prescribed**	**Attitudes**
**Knowledge**	-0.27**	-0.27**	-0.08	-0.17*	0.54**

* P < 0.05

** *P* < 0.01

A moderate positive association was found between pediatricians’ level of knowledge and their attitudes to performing eye tests (their perception that it is part of their job as pediatricians to treat red eye and even to perform eye tests in response to children’s complaints of red eye) (*r* = 0.54, *p* < 0.01). Namely, the higher pediatricians’ level of knowledge the more positive their attitude to treating red eye.

A negative association was found between the level of knowledge and the number of cases in which pediatricians prescribed antibiotics for children with red eye. Namely, the lower the level of knowledge on treating red eye, the higher the number of antibiotic prescriptions given to children.

The research findings indicated that more than half the pediatricians, 56% (*n* = 86), reported that during their residency they had not received the necessary tools for treating pediatric eye disorders. Pediatricians who claimed that they had insufficient tools tended to be younger and to have less experience (see Table 3).

**Table 3:** Difference between pediatricians mean years of experience and age, as related to their view on imparting tools for treating eye disorders in children

		**M**	**SD**	**t**	**df**	***P***
**Age**	**Does not have (*n* = 86)**	43.55	10.72	-2.18	150	0.03
	**Has (*n* = 66)**	47.75	12.94			
**Years of experience**	**Does not have(*n* = 86)**	12.73	11.75	-2.15	150	0.04
	**Has(*n* = 66)**	17.42	15.01			

## Discussion

The current study examined the knowledge level of pediatricians with regard to treating children with red eye, as well as factors affecting the knowledge level and the potential implications of a low level of knowledge. The study found that the respondents’ level of knowledge on treating red eye was moderate. On one hand, most of the respondents knew that the most reasonable diagnosis for a child with red eyes and discharge is viral conjunctivitis and that pain, blurred vision, and a clouded cornea, are symptoms indicative of a more complex systemic problem. Most also knew that red eye, together with other symptoms, might indicate a more severe condition. Hence, it can be assumed that the respondents diagnosed well. Nonetheless, it is notable that some 10% of the respondents could not answer these basic questions correctly. These participants did not differ in terms of age, gender, and years of experience from the rest of the sample. The findings contradict those found in studies conducted in Kenya, Ethiopia, and Jordan [6, 7, 10], which revealed a low level of knowledge among pediatricians treating red eye. A possible reason may be the training program of pediatricians in Israel as well as the training provided on the job.

In contrast, a certain decline in knowledge was found for questions on treating red eye, both regarding the most common cause of red eye and when red eye is a symptom of a more complex condition. Thus, 78.3% of the physicians claimed that the treatment of choice for viral conjunctivitis is a conservative treatment that includes eye flushing and strict hygiene, and 71.7% said that they would refer a child with red eye to an ophthalmologist in any case of disrupted or blurred vision. The suboptimal level of knowledge does not suit the important role assigned to pediatricians as gatekeepers for the early detection and prompt treatment of ocular disorders in children, to prevent lifelong visual impairment.

The research findings indicate several factors that might be related to pediatricians’ level of knowledge on treating red eye among children. Thus, the higher the age and the more years of experience, the lower the physician’s level of knowledge on treating red eye among children. Namely, it seems that it is the more experienced physicians that have a lack of knowledge regarding red eye. A possible explanation of these findings is that the knowledge of veteran physicians regarding red eye is not refreshed. They are not updated and remain “stuck”. Their practice in this area is not based on recent evidence [10].

Interestingly, the current study found no association between the number of cases involving red eye treated by physicians and their knowledge level. This finding is compatible with the previous finding whereby more years of experience do not necessarily translate into more knowledge on red eye [9].

It appears from the research findings that pediatricians’ level of knowledge regarding red eye might have several implications. Thus, the higher physicians’ level of knowledge, the more positive their attitude to treating red eye, namely, they agreed that it is part of their job as pediatricians to treat red eye as well as to test a child’s eyes when encountering a complaint of red eye, similar to research findings from Germany [11]. Compatible with the lack of knowledge, the study indicated that the lower the level of knowledge on treating red eye the higher the number of antibiotic prescriptions given to children. This is concerning because the treatment might be unnecessary and even harmful in cases of sensitivity.

The research findings show that more than half the pediatricians reported that during their residency they had not received the necessary tools for treating pediatric eye disorders. Pediatricians who claimed that they had insufficient tools also tended to be younger and with less experience. This finding seems to contradict the other finding of this study, whereby the higher the physician’s age and years of experience, the lower the level of knowledge on treating red eye in children. This finding may be due to the response bias.

### Research limitations

This study employed a relatively small convenience sample, which limits the generalizability of its results. Thus, the actual knowledge level might be even lower that that found. In addition, in the light of the fact that the response rate in the present study was 76%, response bias cannot be excluded. Moreover, as this study has a survey design, its results may be prone to social desirability bias. Finally, due to the correlational design of the study, causality cannot be determined.

## Conclusions and suggestions

The research findings indicate a moderate level of knowledge among pediatricians, where the issue of treatment is slightly weaker than that of diagnosis. The problem involving lack of knowledge was more conspicuous among the more experienced pediatricians. It appears that knowledge is critical both for readiness to treat red eye and for providing proper treatment.

However, it is clear from our results that more education in this area, particularly in the case of older pediatricians, is still necessary. Identification of dry eye as a common cause of red eye symptoms and more appropriate treatment of dry eye, allergic conjunctivitis, and viral conjunctivitis, are key messages that emerge from this study. Hence, it is necessary to verify that pediatricians receive appropriate tools for treating eye disorders in children and that their knowledge on red eye is refreshed, particularly in the case of more experienced pediatricians.

## Data Availability

The data that support the findings of this study are available from the corresponding author, Dr. Merav Ben Natan, upon reasonable request.
